# Prognostic Factors in Hospitalized COVID-19 Patients at the Start of the Pandemic in Colombia: A Retrospective Cohort Study

**DOI:** 10.7759/cureus.14865

**Published:** 2021-05-06

**Authors:** Jose A Nuñez-Ramos, Elias Forero Illera, Jorge Luis Quintero Barrios, Hugo Andres Macareno Arroyo, Didier Johanna Larios Sanjuan, Maria Carolina Manzur Barbur, Ana Milena Mejia Sanjuanelo, Mauro Duvan Mendoza Quevedo, Diego Fernando Viasus-Perez

**Affiliations:** 1 Health Sciences Division, Universidad del Norte, Barranquilla, COL; 2 Internal Medicine, Hospital Universidad del Norte, Barranquilla, COL; 3 Rheumatology, Hospital Universidad del Norte, Barranquilla, COL; 4 Pulmonology, Hospital Universidad del Norte, Barranquilla, COL; 5 Infectious Diseases, Hospital Universidad del Norte, Barranquilla, COL

**Keywords:** covid-19, sars cov-2, covid prognosis, disease mortality, tocilizumab

## Abstract

Introduction

Different factors are critical when assessing COVID-19 mortality, and can explain why severity differs so widely among populations. However, there is little information regarding prognostic factors and mortality in COVID-19 from Latin American countries.

Objectives

To determine prognostic factors in hospitalized COVID-19 patients and to evaluate the impact of tocilizumab use in patients with hyperinflammatory syndrome and severe disease defined by the National Early Warning Score 2 (NEWS2) with a value greater than or equal to seven points.

Materials and methods

This retrospective cohort study included hospitalized COVID-19 patients from May to July 2020. A multivariate logistic regression analysis was performed to determine independent factors associated with mortality.

Results

A total of 136 patients required hospital admission. In-hospital mortality was 39.7%. Mortality was observed to be potentiated by older age, LDH serum levels and the presence of type 2 diabetes mellitus. Lymphopenia and lower PaO2/FiO2 ratio were more common in these patients. Similarly, patients who died were classified more frequently with severe disease. The independent factors associated with in-hospital mortality were age greater than 65 years, type 2 diabetes mellitus, NEWS2 greater than or equal to seven points and LDH greater than 400U/L. The use of Tocilizumab alone was not related with decreased in-hospital mortality. Subgroup analysis performed in patients with hyperinflammation and severe disease showed similar results.

Conclusions

COVID-19 mortality in hospitalized patients was high and mainly related with older age, comorbidities, LDH and the severity of disease at hospital admission.

## Introduction

The coronavirus disease 2019 (COVID-19) was classified as a pandemic in February 2020 [1]. Approximately 5%-10% of patients require hospital admission due to the development of moderate to severe disease. Importantly, critically ill patients have higher mortality ranging from 20% to 60% [2]. Different factors are critical when assessing COVID-19 mortality, and these factors can explain why severity differs so widely among people and countries [3]. The risk of mortality is higher in older populations and in patients with comorbid conditions [4]. In addition, it has been proposed that an inadequate inflammatory response is one of the main causes of mortality in COVID-19 patients, leading to an important interest in order to identify treatments that can modulate this augmented response [5].

Dexamethasone has demonstrated a clinical impact reducing mortality in hypoxemic patients that require hospital admission [6]. Moreover, interleukin 6 (IL-6) receptor blockade seems to be a promising treatment for a specific group of COVID-19 patients. However, study findings are controversial [7-12].

There is little information regarding mortality and prognostic factors in COVID-19 from Latin América. Thus, this study aimed to determine prognostic factors in Colombian hospitalized COVID-19 patients and to evaluate the impact of tocilizumab use in patients with hyperinflammatory syndrome and severe disease defined by the National Early Warning Score 2 (NEWS2) greater than or equal to seven points.

## Materials and methods

This is a retrospective cohort study of hospitalized COVID-19 patients admitted, from May 20th to July 31st in a convenience sample, to the Hospital Universidad del Norte, a 136-bed university hospital in Soledad, Colombia. Clinical-suspected and laboratory-confirmed COVID-19 patients were included. A clinical-suspected COVID-19 case was defined as a patient with upper or lower respiratory tract symptoms with compatible radiological computerized tomography (CT) lung scan (peripheral ground-glass opacities) and no other etiologies documented. Laboratory-confirmed COVID-19 case was defined as a clinical-suspected COVID-19 case with positive results in real-time polymerase chain reaction (RT-PCR) for severe acute respiratory syndrome coronavirus 2 (SARS-CoV-2). RT-PCR for SARS-CoV-2 was performed from respiratory samples in all patients. The study was approved by the Institutional Ethics Committee (ID213-2020).

Patients were divided into two groups: (i) patients who survived; and (ii) patients who died. The independent variables were clinical characteristics and laboratory values. The treatment of COVID-19 pneumonia was according to institutional protocol and included antibiotics, oxygen, steroids and tocilizumab. Regarding tocilizumab, it was used in patients with hypoxemia and elevated markers of inflammation. Tocilizumab was not administered to patients with suspicion of concomitant bacterial infection. The administered intravenous dose was 400 mg for individuals less than 75 kg and 600 mg for those greater than or equal to 75 kg. All tocilizumab doses were given within the first 24 hours of hospital admission.

To address illness severity we used the NEWS2 [13]. Severe disease was defined as NEWS2 score of greater than or equal to seven points. Hyperinflammation was defined according to recent validated criteria in COVID-19 infection [5] that included two or more of these laboratory findings: ferritin >700 ng/dl, c-reactive protein >80 mg/dl, d-dimer >1,000 ug/L, lymphocyte <900 cells/ul, or lactate dehydrogenase (LDH) >400 U/L. 

Statistical analysis

The categorical variables were reported as frequencies and percentages and the continuous variables as median and interquartile range (IQR). Normal distribution was tested. The Mann-Whitney U-test was used for comparing continuous variables and chi-square or Fisher's test for qualitative variables. To determine independent factors associated with in-hospital mortality, a multivariate analysis was performed. The model included important clinical variables, including tocilizumab, and those with significant difference between groups. Results of multivariate analyses were reported as odds ratio (OR) and 95% confidence intervals (CI). The goodness of fit of the multivariate model was assessed by the Hosmer-Lemeshow test and the accuracy by the area under receiver operating characteristic (AUROC) curve. We performed subgroup analyses according to the presence of hyperinflammation and severe disease using NEWS2 ≥7. The threshold for statistical significance was defined as a two-tailed p < 0.05. All analyses were performed using the SPSS software, version 25 (IBM Corp., Armonk, NY). 

## Results

During the study period, 136 patients with COVID-19 were admitted to the hospital. Regarding RT-PCR results, 113 (83.1%) were classified as laboratory-confirmed COVID-19 and 21 (16.9%) as clinical-suspected COVID-19.

Characteristics of the cohort

Clinical features and laboratory findings of all patients are shown in Table 1. Among the total population, 59 (43.4%) patients were older than 65 years and were mostly males. The main comorbid condition was hypertension, followed by type 2 diabetes mellitus. The most common symptoms were cough, fever and dyspnea. Diarrhea and gastrointestinal manifestations were present in 10% of patients. Most patients had altered laboratory findings, such as lymphopenia, elevated ferritin, LDH, C-reactive protein and d-dimer. Regarding lung CT-scan, 90% of patients were classified as having typical findings of SARS-CoV2 pneumonia (CO-RADS 4 and 5). Once admitted, 41 (30.1%) received antiviral therapy (hydroxychloroquine, oseltamivir, or lopinavir/ritonavir). Remdesivir was not an option considering it is not available in Colombia. Regarding interventions, 61 patients (44.9%) received tocilizumab and 85 (66.4%) corticosteroids (methylprednisolone or dexamethasone). In-hospital mortality was 39.7%, accounting for 54 patients. Ninety (66.2%) patients required ICU admission and almost 10% were intubated at emergency admission. 

**Table 1:** Baseline characteristics and according to survival in hospitalized COVID-19 patients. Values are median and interquartile range for continuous variables. Frequencies in #(%) for categorical. NEWS2: National Early Warning Score 2; LDH: lactate dehydrogenase; COPD: chronic obstructive pulmonary disease; ICU: intensive care unit.

Clinical features	All patients, n = 136	Survivors, n = 82	Non-survivors, n = 54	p-value
Age, years	62 (51-74)	57 (45-66)	68 (60-79)	<0.001
18-40 years	18 (13.2)	16 (19.5)	2 (3.7)	
41-65 years	60 (44.1)	44 (53.7)	16 (29.6)	
>65 years	58 (42.6)	22 (26.8)	37 (68.5)	<0.001
Male	73 (53.7)	46 (56.1)	27 (50)	0.485
Hypertension	55 (40.4)	29 (35.4)	26 (48.1)	0.137
Type 2 diabetes	42 (30.9)	20 (24.4)	22 (40.7)	0.001
Cardiovascular disease	4 (2.9)	1 (1.2)	3 (5.6)	0,301
COPD/asthma	11 (8)	4 (4.8)	7 (12.9)	0,82
Days of symptoms	7 (4-8)	7 (5-9)	4 (3-7)	0.023
Presence of fever	100 (73.5)	66 (80.5)	34 (63)	0.978
Cough	93 (68.4)	56 (68.3)	37 (68.5)	0.354
Dyspnea	101 (74.3%)	52 (85.2)	49 (65.3)	0,008
Diarrhea	17 (12.5)	9 (14.8)	8 (10.7)	0.473
Admission laboratory findings				
PaO2/FiO2 ratio	203 (110-338)	247 (155-360)	158 (51-283)	0.001
D-dimer level (ug/L)	700 (300-2150)	660 (300-1400)	980 (400-6500)	0,037
Ferritin level (ng/dl)	1058 (464-1650)	1051 (485-1643)	1144 (457-1650)	0.870
LDH level (u/L)	411 (330-560)	380 (288-433)	549 (410-662)	<0.001
C-reactive protein level (mg/dl)	161 (125-174)	159 (104-177)	162 (140-177)	0.463
Lymphocytes (cells/ul)	810 (552-1220)	965 (607-1300)	690 (487-1115)	0.035
Aspartate aminotransferase (U/L)	50 (36-77)	45 (31-72)	56 (38-80)	0.108
Alanine aminotransferase (U/L)	40 (25-66)	40 (27-71)	43 (24-61)	0.469
Creatinine (mg/dl)	1.1 (0.9-1.5)	1.0 (0.9-1.3)	1.4 (1.0-2.2)	<0.001
NEWS2 score	6 (4-8)	6 (4-7)	8 (5-10)	<0.001
Nasal cannula	40 (29.4%)	16 (26.2)	24 (32)	0.463
Non-rebreather mask	51 (37.5%)	26 (31.7)	25 (46.3)	0.086
Orotracheal intubation at admission	13 (9.6)	2 (2.4)	11 (20.4)	0.001
Interventions				
Antivirals	41 (30.1%)	23 (28)	18 (33.3)	0.534
Corticosteroids	85 (66.4)	50 (64.9)	35 (68.6)	0.665
Tocilizumab	61 (44.9)	40 (48.8)	21 (38.9)	0.256
ICU admission	90 (66.2)	41 (50)	49 (90.7)	<0.001

Factors associated with mortality

Patients who survived were compared with patients who died (Table 1). Patients who died were older and had more frequently type 2 diabetes mellitus. Similarly, higher serum levels of LDH, creatinine and d-dimer were present in patients who died. Lower PaO2/FiO2 ratio and absolute lymphocyte count were also most common in these patients. Patients who died were more frequently classified with severe disease at admission, as evaluated by NEWS2 score. There were no differences in the use of tocilizumab and corticosteroids between survivors and non-survivors

A logistic regression analysis was conducted to determine independent factors associated with in-hospital mortality (Table 2). The independent variables associated with in-hospital mortality were age >65 years, type 2 diabetes mellitus, NEWS2 ≥ 7 and LDH >400U/L. The use of tocilizumab was not related with in-hospital mortality. The Hosmer-Lemeshow test result of the model was 0.598 and the AUROC was 0.84 (CI 95%, 0.77-0.91). 

**Table 2:** Logistic regression analysis of factors associated with mortality. Other variables included in the multivariate model were hypertension, ferritin, d-dimer and c-reactive protein. NEWS2: National Early Warning Score 2; LDH: lactate dehydrogenase

	Adjusted OR (IC95%)	p-value
Age ≥65 years	6.0 (2.4-15.1)	<0.001
Type 2 diabetes mellitus	3.0 (1.0-9.0)	0.045
NEWS2 ≥7	3.5 (1.4-8.7)	0.007
LDH >400 U/L	5.7 (2.1-15.2)	<0.001
Tocilizumab	0.5 (0.1-1.6)	0.273
Corticosteroids	1.3 (0.4-4.3)	0.613

Subgroup analyses

Subgroup analyses were performed in patients with hyperinflammatory syndrome and with severe disease. In the multivariate regression analyses, the factors associated with mortality were similar to those in the general population. Tocilizumab was not a protective factor related with in-hospital mortality neither in hyperinflammatory syndrome (OR 0.50, CI 95% 0.15-1.6; p = 0.25) nor in NEWS≥7 subgroups (OR 0.27, CI 95% 0.04-1.9; p = 0.19).

## Discussion

In this retrospective cohort study in hospitalized patients with COVID-19, mortality was high and mainly related with older age, type 2 diabetes mellitus, high levels of LDH and the severity of disease at hospital admission. The use of tocilizumab was not related with better prognosis, even in patients with severe disease and patients with hyperinflammatory syndrome.

The present results are consistent with data from studies that evaluated prognostic factors in hospitalized patients with COVID-19. In the univariate analysis of this study, age, type 2 diabetes mellitus, dyspnea, PaO2/FiO2 ratio, serum levels of d-dimer, LDH and creatinine, and lymphocyte count were factors associated with a higher risk of in-hospital mortality. In this regard, there is evidence that advanced age, comorbidities, abnormal inflammatory and organ injury circulating biomarkers were more frequently found in patients with an adverse clinical outcome [4]. Thus, clinical history and laboratory profile may help identify patients with a higher risk of in-hospital mortality. In fact, most of these factors have been included in severity scores developed to evaluate the risk of complications in COVID-19 patients [13,14].

We documented a higher mortality among hospitalized COVID-19 patients (39.7%) compared with that found in other studies (9%-25%) [15]. In addition, our cohort also had higher frequency of ICU admission (66.2%). Factors such as age, sex and comorbidities were not different in our cohort compared with that reported from other COVID-19 patients. However, some factors can explain these poor outcomes in our population. First, Consuegra et al [16] reported the high frequency of early mortality (≤24 hours of hospital admission) in COVID-19 patients. Early deaths were explained by a more severe disease at hospital admission. Delay in seeking early medical care may be a factor favoring the unacceptably high frequency of mortality during the first 24 hours of hospital admission in these patients. Second, a study documented that the risk of dying for COVID-19 among confirmed cases in Colombia was higher in people with subsidized health insurance regime and in people living in very low socioeconomic strata [17]. In this regard, the hospital area of ​​influence, located in the city of Soledad, mainly serves a population with a high level of poverty (78.1% between strata 1 and 2). Finally, when health system resources were limited, patients with the more severe illness were hospitalized and those with mild illness were transferred to other institutions.

Regarding treatment, studies evaluating interleukin 6 (IL-6) receptor blockade in COVID-19 patients have documented controversial results. Multiple observational studies have shown a discrete mortality reduction in patients with moderate to severe pneumonia due to COVID-19 [18-20]. Similarly, Salama et al. [21] published a multicenter randomized double-blind, placebo-controlled clinical trial performed in patients not receiving mechanical ventilation. Investigators found a reduction in the composite outcome of progression to mechanical ventilation and mortality in those using tocilizumab. Conversely, comparable to our findings, Stone et al. [12] published a randomized double-blind, placebo-controlled clinical trial conducted in patients with COVID-19 pneumonia along with hyperinflammatory syndrome and/or oxygen requirement. This study did not find differences in the mechanical ventilation need or mortality with the use of tocilizumab. Recently, the REMAP-CAP trial was published reporting a benefit in in-hospital survival and organ support-free days using tocilizumab and sarilumab [22]. It can be theorized that studies of immunomodulatory treatments have been unsuccessful as a result of the incomplete understanding of the immune response during COVID-19, and the heterogeneity of studies population in terms of age, comorbidities and disease severity. Thus, current guidelines recommend tocilizumab in a specific group of hospitalized adults with progressive severe or critical COVID-19 who have elevated markers of systemic inflammation and who had no response to steroids [23]. Future studies should evaluate if patients have distinct laboratory patterns that reflect various clinical complications requiring different therapy approaches [24].

The strengths of this study are the comprehensive clinical data gathered. This information allowed us to identify specific subgroups of patients. In addition, we performed a multivariate analysis to control confounding factors. The accuracy of the multivariate model was good, as evaluated by the area under the ROC curve. However, our study has some limitations that should be acknowledged. The selection bias restricts the results analysis and the limited sample of patients undermines the power of the study. Moreover, there is no standardized criteria for hyperinflammatory syndrome in COVID-19 and almost every study has a different definition. However, we used the most recent validated COVID-19 hyperinflammatory syndrome criteria in our analysis [5]. Another important limitation was that corticosteroids were not used in all of our patients, because it was not a standard therapy for hospitalized COVID-19 patients during the study period. Finally, some patient features that can be related with outcomes, such as obesity, socioeconomic level, or ethnicity were not recorded.

## Conclusions

In conclusion, COVID-19 mortality was high during the first pandemic wave. The main factors related with mortality were older age, comorbidities as diabetes mellitus, high levels of LDH and the severity of disease at hospital admission according to NEWS2. Tocilizumab had no relation with mortality in global population and in subgroups. Studies should assess if, among COVID-19 patients, there are different inflammatory patterns, such as hyperinflammation, and if different treatment approaches may improve outcomes in specific subgroups of patients.
